# Quantitative Evaluation of Native Protein Folds and Assemblies by Hydrogen Deuterium Exchange Mass Spectrometry (HDX-MS)

**DOI:** 10.1007/s13361-018-2070-3

**Published:** 2018-10-02

**Authors:** Matthew J. Harris, Deepika Raghavan, Antoni J. Borysik

**Affiliations:** Department of Chemistry, King’s College London, Britannia House, London, SE1 1DB UK

**Keywords:** Hydrogen deuterium exchange mass spectrometry, Protein structure

## Abstract

Hydrogen deuterium exchange mass spectrometry (HDX-MS) has significant potential for protein structure initiatives but its relationship with protein conformations is unclear. We report on the efficacy of HDX-MS to distinguish between native and non-native proteins using a popular approach to calculate HDX protection factors (PFs) from protein structures. The ability of HDX-MS to identify native protein conformations is quantified by binary structural classification such that merits of the approach for protein modelling can be quantified and better understood. We show that highly accurate PF calculations are not a prerequisite for HDX-MS simulations that are capable of effectively discriminating between native and non-native protein folds. The simulations can also be performed directly on unique structures facilitating high-throughput evaluation of many alternate conformations. The ability of HDX-MS to classify the conformations of homo-protein assemblies is also investigated. In contrast to protein monomers, we show a significant lack of correspondence between the simulated and experimental HDX-MS data for these systems with a subsequent decrease in the ability of HDX-MS to identify native states. However, we demonstrate surprisingly high diagnostic ability of the simulated data for assemblies in which a significant proportion of the individual chains occupy protein-protein interfaces. We relate this to the number of peptides that can sample alternate subunit orientations and discuss these observations within the larger context of applying HDX-MS to evaluate protein structures.

**Electronic supplementary material:**

The online version of this article (10.1007/s13361-018-2070-3) contains supplementary material, which is available to authorized users.

**Figure:**
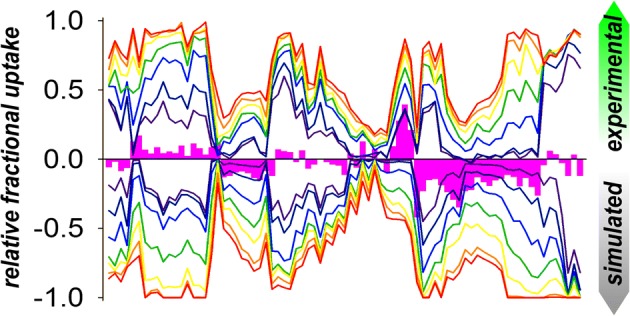
Graphical Abstract

## Introduction

Hydrogen deuterium exchange mass spectrometry (HDX-MS) reports on time-dependent changes in the deuterium uptake of a protein in D_2_O solvent with a structural probe at virtually every amino acid along the protein backbone [1–3]. Despite many advantages of HDX-MS including speed and sensitivity, the method is normally limited to providing qualitative insight into protein conformations. Protein structures are typically required to inform on experimental outputs but the use of HDX-MS to determine protein structures is something of a novelty. We recently demonstrated the potential for simulating the HDX-MS patterns of proteins to elucidate the structures of hetero-protein assemblies [4]. Here, HDX protection factors (PFs) were estimated from atomic coordinates and then used to modify the chemical exchange rates of residues to calculate the isotope uptake of each peptide. The approach facilitated the high-throughput ranking of docking poses based on pairwise comparisons with experimental data. Importantly, it permitted the quantitative discrimination of different poses without the need for additional processing or user interpretation.

The potential for determining native protein folds by HDX-MS is another exciting application of the technique. Accurately predicting protein exchange rates remains a significant challenge although the ability of predictive tools to discriminate between native and non-native folds by HDX-MS has not been previously investigated or quantified [5–8]. Here, we extend our previous work on HDX-MS protein modelling to investigate the performance of these methods to identify native protein folds and the conformations of homomeric protein assemblies. We show that the HDX-MS patterns of proteins simulated directly from their atomic structures are sufficiently accurate to discriminate between native and non-native protein folds. In contrast, the simulated HDX-MS profiles of homo-protein complexes are shown to correspond poorly with their respective experimental outputs. Surprisingly, the capacity to discriminate between native and non-native quaternary structures of protein complexes is high for protein assemblies in which each subunit has multiple interchain contacts. We relate this to an increase in the number of peptides that can sample alternate chain orientations in these systems. Taken together, these data add to our understanding of the use of HDX-MS for structural evaluation and provide an important foundation on which future developments in the area can be built.

## Methods

### Mass Spectrometry

HDX-MS experiments were performed on a Synapt G2Si HDMS coupled to an Acquity UPLC M-Class system with HDX and automation (Waters Corporation, Manchester, UK). Human alpha lactalbumin (Athens Research and Technology Inc., Athens, USA), enolase from baker’s yeast (Sigma-Aldrich Ltd., Dorset, UK) and serum amyloid P component (SAP) from human serum (Merck Chemicals Ltd., Nottingham, UK) were purchased as lyophilised powder, and barnase was prepared in-house. The isotope uptake of each protein was determined using a continuous labelling workflow at 20 °C. Each protein was dissolved in buffer E (10 mM potassium phosphate pH 7.0) to a final concentration of 5–10 μM. Isotope labelling was initiated by diluting 5 μl of each protein into 95 μl of buffer L (10 mM potassium phosphate in D_2_O pD 6.6) for various time points. Aliquots of each reaction were taken and quenched by diluting in equal volumes of ice-cold 2% formic acid. Human alpha lactalbumin was quenched in an equal volume of 10 mM phosphate buffer containing 0.4 M tris(2-carboxyethyl)phosphine hydrochloride (Bertin Pharma, Bretonneux, France) and 1.5% HCl to promote pepsin digestion by reduction of disulphide bonds and barnase quench solutions contained 4 M urea. Proteins were digested online with a Waters Enzymate BEH pepsin column at 20 °C. The coverage and redundancy of alpha lactalbumin and barnase digestion were enhanced by increasing the column pressure to 7000 psi with the aid of a back pressure regulator (Waters Corporation). Peptides were trapped on a Waters BEH C18 VanGuard pre-column for 3 min at a flow rate of 200 μl/min in buffer A (0.1% formic acid ~ pH 2.5) before being applied to a Waters BEH C-18 analytical column. Peptides were eluted with a linear gradient of buffer B (0.1% formic acid in acetonitrile ~ pH 2.5) at a flow rate of 40 μl/min. All trapping and chromatography were performed at 0.5 °C to minimise back exchange. MS data were acquired using an MS^E^ workflow in HD mode with extended range enabled to reduce detector saturation and maintain peak shapes and all labelling time points were obtained in triplicate. The MS was calibrated separately against NaI and the MS data were obtained with lock mass correction using Leu-enkephalin. Peptides were assigned with the ProteinLynx Global Server (PLGS, Waters Corporation, Manchester, UK) software and the isotope uptake of each peptide determined with DynamX v3.0. The isotope uptake of each peptide was corrected for back/in exchange according to methods outlined by Zhang [1]. Fully deuterated protein samples were prepared by dissolving lyophilised samples in buffer L; each sample was then sterilised using a 0.2-μm syringe filter prior to incubation at 37 °C for at least 3 weeks. The isotope uptake of each peptide is reported as the relative fractional uptake (RFU) which is the observed mass shift of a peptide normalised to the maximum possible change in mass.

### Simulating Protein HDX-MS Patterns

HDX protection factors (PFs) were estimated according to near-contacts criteria and hydrogen bonding as previously described where the protection of residue *i* (${\mathrm{lnP}}_i^{\mathrm{sim}}$) is expressed as the number of heavy atoms (${N}_i^C$) and hydrogen bond acceptors (${N}_i^H$) within defined distance cutoffs from the backbone amide each weighted by an empirically determined scaling term (*β*) (Eq. 1) [4, 5]:

1 $$ \ln {\mathrm{P}}_i^{\mathrm{sim}}={N}_i^C{\beta}_C+{N}_i^H{\beta}_H $$

When compared to experimental data previously obtained by NMR, Eq. 1 significantly overestimates the PFs of backbone amides [9]. To account for this discrepancy, a separate exclusion parameter (excl) was introduced that allowed the outputs to be rescaled by omitting the contribution of all heavy atoms from the contact calculations of user-defined residues: where excl = 0 reports all heavy atoms for PF calculations of residue *i*; excl = 1 omits the atoms of residue *i*; excl = 2 omits the atoms of residue *i* and immediately adjacent residues and so on. In addition to this, a smoothing function was also introduced for atom counting within the cutoff distance, where dist(*h*, *O*) and dist(*n*, heavyAtom) are the linear distances relating to the respective hydrogen bond and contact calculations and hcut and heavycut are the respective cutoff distances of 2.4 and 6.5 Å (Supporting Information, Fig. S1, Eq. 2) [10]:

2 $$ \ln {\mathrm{P}}_i^{\mathrm{sim}}=\frac{\beta_H}{1+{e}^{10\mathrm{dist}\left(h,O\right)-\mathrm{hcut}}}+\frac{\beta_C}{1+{e}^{5\mathrm{dist}\left(n,\mathrm{heavyAtom}\right)-\mathrm{heavycut}}} $$

PFs were simulated directly from the corresponding crystal structures (1A4V, 1A2P, 1SAC and 3ENL) with missing structure built using Modeller [11–15]. In the case of alpha lactalbumin, PFs were also calculated from a protein ensemble generated by molecular dynamics (MS) simulations of 1A4V in explicit water. MD simulations were performed using the OPLS/AA force field implemented within GROMACS 4.6.7 [16]. Production MD simulations were carried out at 300 K for 100 ns following energy minimisation and extensive solvent equilibration. One hundred structures were taken along the 100-ns trajectory and protection factors expressed as the average values taken across all conformations. Alpha lactalbumin and barnase decoy sets were prepared using 3DRobot with the output set to 1000 structures [17]. A range of enolase and SAP decoys were prepared using a local installation of SymmDock V1.0 without constraints yielding ca. 10,000 and 5000 transformants for enolase and SAP respectively [18]. Transformants were then refined on a local installation of SymmRef V1.2 using the recommended settings to remove steric clashes and allow for backbone and sidechain flexibility [19].

The simulated PFs were used to generate HDX-MS patterns of each protein using an in-house script implemented within MATLAB. In the case of enolase and SAP, the PFs of each residue were taken as the average across all protein chains. The code takes as input the protein sequence, experimental peptide list of a protein and the start and end positions of each peptide along with the experimental temperature and pD. It then calculates the intrinsic chemical exchange rates (*k*_int_) of each backbone amide proton according to previously defined near-neighbour effects using the modified exchange factors for acidic residues [20, 21]. The intrinsic exchange rates and PFs are then used to determine the observed exchange rates (*k*_obs_) for each residue according to Eq. 3. The isotope uptake of each peptide is then calculated from the following polyexponential function, where *D*_t_ is the total number of deuterium atoms incorporated into the peptide at time *t*, *N* is the total number of exchangeable positions and *k*_*i*_ is the observed hydrogen exchange rate constant of residue *i* (Eq. 4):

3 $$ {k}_{\mathrm{obs}}=\frac{k_{\mathrm{int}}}{\mathrm{PF}} $$

4 $$ {D}_t=N-\sum \limits_{i-1}^N\exp \left(-{k}_it\right) $$

Proline residues were discounted along with amino-terminal groups to ensure that the simulated RFU calculations were in line with experimental outputs processed by DynamX.

### Expression and Purification of Barnase

Unless stated otherwise, all chemicals were purchased from Fluorochem Ltd., Derbyshire, UK, Sigma-Aldrich Ltd., Dorset, UK, or VWR International Ltd., Leicestershire, UK. Overexpression of wild-type barnase (*Bacillus amyloliquefaciens* ribonuclease) was directed from the plasmid pTZ416 under the control of the alkaline phosphatase promotor and was kindly provided by Prof Teikichi Ikura (Tokyo Medical and Dentistry University, Japan) [22]. The plasmid was transformed into BL21(DE3)pLysS cells and plated onto LB agar plates containing ampicillin (50 mg/ml) and chloramphenicol (34 mg/ml). A single colony was used to inoculate 50 ml LB containing ampicillin and chloramphenicol and incubated overnight at 37 °C with agitation at 220 rpm; 1.2 ml of the pre-culture was then used to inoculate 200 ml low-phosphate media containing ampicillin and chloramphenicol and incubated overnight at 30 °C with agitation at 110 rpm. The low-phosphate media was prepared as follows. For 1 l low-phosphate media, 0.4 g casamino acids was added to 900 ml H_2_O and autoclaved. To this, 100 ml 10 × concentrate filter sterilised MOPS (3-(*N*-morpholino)propanesulfonic acid) was added containing 10 ml 20% glucose, 0.1 ml 1 M neutral phosphate buffer, 1 ml of 20 mg/ml adenine, 50 μl 10 mg/ml thiamine, 1 ml 50 mg/ml ampicillin and 1 ml 34 mg/ml chloramphenicol. The concentrated MOPS buffer contained 0.4 M MOPS, 42 mM tricine, 95 mM NH_4_Cl, 2.8 mM K_2_SO_4_, 5.3 mM MgCl_2_, 0.5 M NaCl, 5 mM CaCl_2_ and 0.1 M FeSO_4_ adjusted to pH 7.4 with NaOH which was then filter sterilised. Immediately prior to use, 10 μl micronutrients was added to the MOPS buffer which contained 3 mM ammonium molybdate, 64 mM cobalt chloride, 80 mM manganese chloride, 0.4 M boric acid, 16 mM copper sulphate and 11 mM zinc sulphate sterilised by filtration. The 1 M neutral phosphate buffer contained 0.5 M Na_2_HPO_4_ and 0.5 M NaH_2_PO_4_ which was then autoclaved. After overnight incubation, 11 ml acetic acid was added to the cell culture and left mixing for 20 min at 4 °C to promote the release of barnase into the media by osmotic shock. The cells were then centrifuged at 7500 rpm for 15 min and the supernatant retained for purification following vacuum filtration through a 0.22-μm filter. Barnase was then equilibrated against two column volumes of dialysis buffer of 50 mM TrisHCl (tris(hydroxymethyl)aminomethane hydrochloride) pH 8.0 before purification by size exclusion chromatography on a Superdex 75 10/300 GL column (GE Healthcare Life Sciences, Little Chalfont, UK). The purification and identity of barnase were confirmed by SDS/PAGE electrophoresis and mass spectrometry.

### Evaluation of HDX-MS Simulations to Identify Native Structures

The ability of the HDX-MS simulations to discriminate between native and non-native protein structures was quantified from the associated receiver operator characteristic (ROC) plots of a binary classification test. The RMSE of each HDX-MS simulation was obtained by pairwise comparison with the associated experimental outputs across all peptides and labelling time points. The RMSD of each decoy was determined by alignment with the relevant native crystal structure using the McLachlan algorithm implemented on a locally installed copy of ProFit v3.1 with decoys having an RMSD ≤ 2.5 Å classified as native [23, 24]. A ROC plot was then generated for each dataset using SigmaPlot 13.0 (Systat Software Inc., London, UK) and the ability of the HDX-MS simulations to identify native structures determined from the area under the curve (AUC) where values > 0.9 were considered excellent, > 0.8 good, 0.6–0.8 poor to fair and below 0.6 failed.

## Results and Discussion

**Figure 1:**
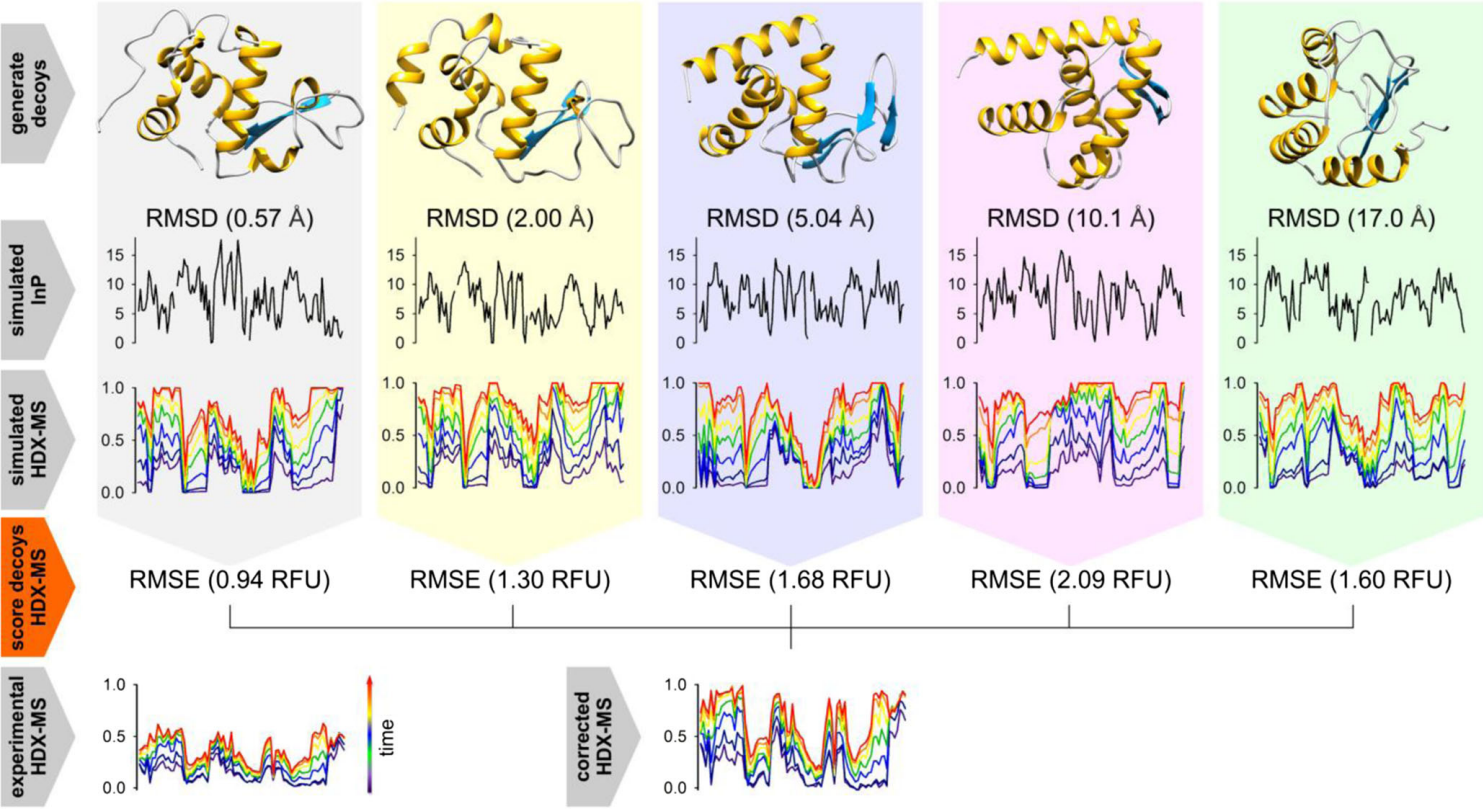
Outline of the HDX-MS simulation workflow and analysis: A set of decoys were first prepared for each protein and the RMSD of each decoy determined by alignment with the native structure. **(top row)** Five example decoys are shown for alpha lactalbumin along with their corresponding RMSD. **(second row)** PFs simulated directly for each decoy according to Eq. 1. **(third row)** The PFs were used to modify the chemical exchange rates and the isotope uptake of each residue determined and projected onto an experimental peptide list to generate a library of simulated HDX-MS profiles. **(fourth row)** The library of HDX-MS simulations was then compared to that of experimental HDX-MS data to obtain the RMSE of each simulation as shown. **(bottom row)** Prior to alignment with the simulated HDX-MS data, all experimental outputs were first corrected for extraneous exchange. Following this process, the simulated HDX-MS profiles were then ranked according to their RMSE with the experimental outputs and their ability to identify native structures evaluated based on their performance in binary structural classification

Many different methods have been developed to estimate the HDX behaviour of proteins but the capacity of these approaches to discriminate between native and non-native states by HDX-MS has not been previously tested or quantified. The ability of HDX-MS to identify native protein folds was evaluated with alpha lactalbumin and barnase with the PFs of these proteins simulated according to Eq. 1 after minor optimisation (Fig. S1, “Methods”) [5]. The PFs were used to modify the chemical exchange rates of these proteins from which the isotope uptake of each residue was determined and projected onto experimental peptide lists to simulate HDX-MS outputs (“Methods”). The ability of the HDX-MS simulations to discriminate between native and non-native folds was evaluated using decoy sets of 1000 different protein conformations. HDX-MS data was simulated for each decoy generating a library of HDX-MS profiles which were ranked according to their correspondence with experimental data obtained in-house (Fig. 1, “Methods”). A binary classification test was then performed to evaluate the efficacy to which the HDX-MS simulations could discriminate between native and non-native protein folds. The diagnostic ability of the simulated HDX-MS profiles was quantified from the area under the curve (AUC) of the associated ROC plots which is a measure of the success rate of correctly classifying structures selected at random (“Methods”).

**Figure 2:**
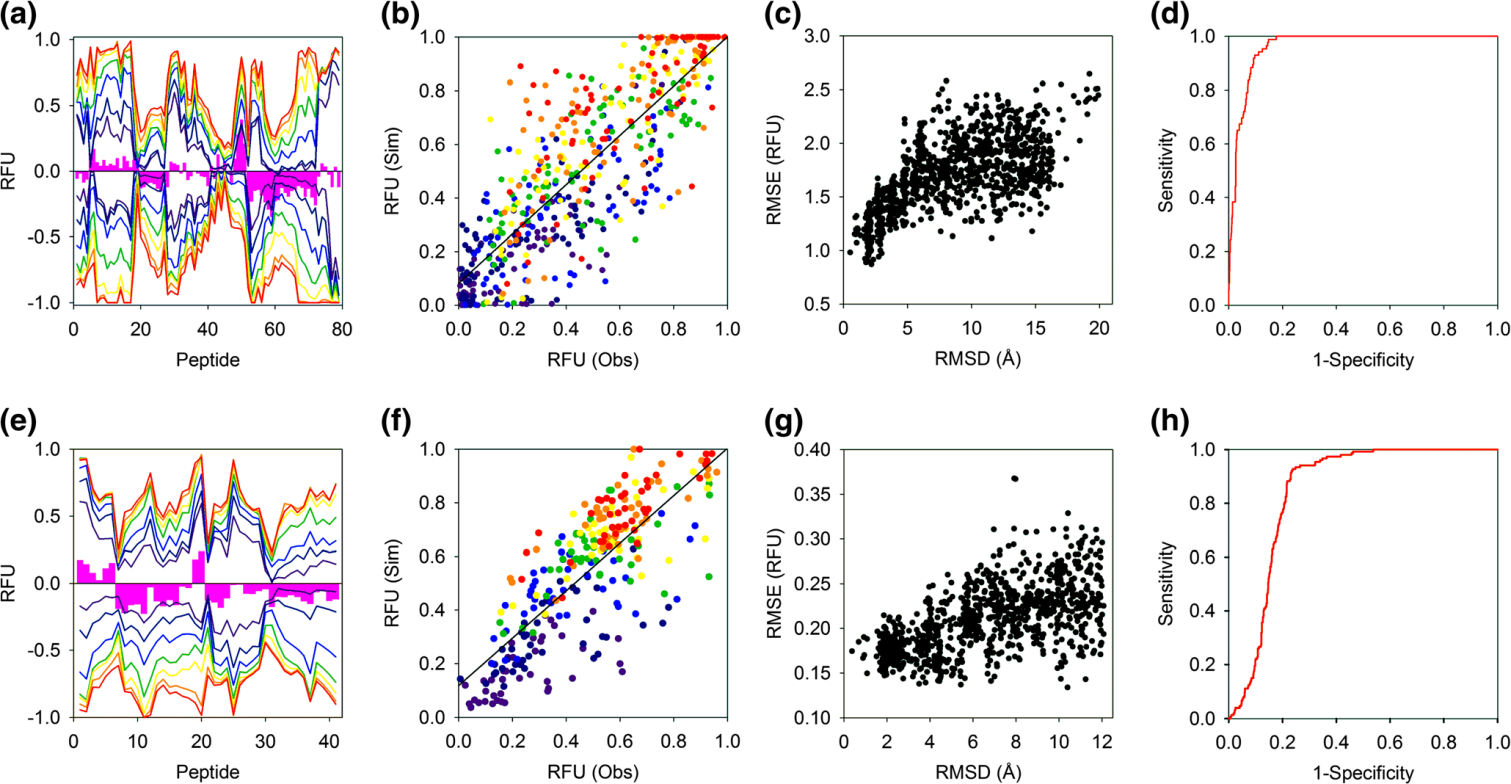
Native folds of alpha lactalbumin and barnase investigated by HDX-MS: (**a**, **e**) Mirror plots comparing experimental (positive) and simulated (negative) HDX-MS outputs. Experimental data were acquired at 0.25, 1, 5, 20, 60, 240 and 480 min at 293.15 K (coloured dark blue through red respectively). The pink bars denote the time-averaged difference in RFU between the experimental and simulated data and are shown to highlight areas of significant change. (**b**, **f**) Scatterplot comparing observed and simulated HDX-MS data of all RFU time points with different labelling times coloured as in (**a**). (**c**, **g**) The relationship between the RMSE and RMSD of 1000 decoys. The RMSE was calculated by pairwise comparison of the simulated and experimental HDX-MS data and the RMSD determined by alignment with the crystal structure. (**d**–**h**) ROC plots demonstrating the ability of the HDX-MS simulations to classify protein structures. Decoys with an RMSD ≤ 2.5 Å with the crystal structure were classified as native. Alpha lactalbumin and barnase data are shown in the upper and lower four figures, respectively

**Figure 3:**
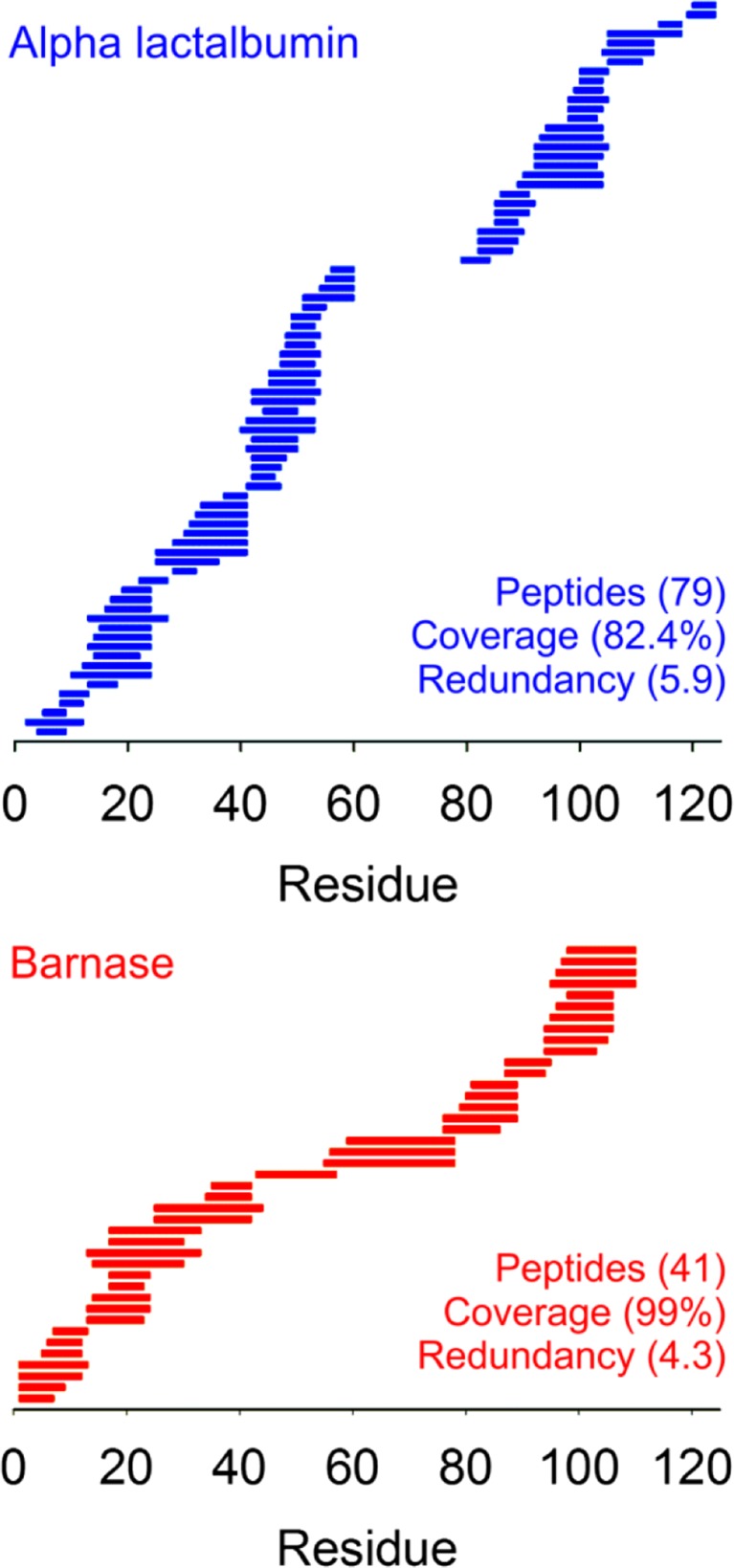
peptide maps of alpha lactalbumin and barnase: The peptide maps of alpha lactalbumin (blue) and barnase (red) that comprise the HDX-MS data of these proteins are shown along with the respective number of peptides, coverage and redundancies. The ~ 20 residue region missing from the alpha lactalbumin data spans two of the four disulphide bonds of the protein

HDX-MS data simulated for the native states of alpha lactalbumin and barnase correlated surprisingly well with experimental outputs of the proteins. For alpha lactalbumin, the experimental and simulated outputs are practically identical over the first ~ 45 peptides with the accuracy of the simulation only breaking down marginally toward the C-terminal end of the protein. The correspondence between the experimental and simulated data of alpha lactalbumin and barnase is comparable with respective RMSE of 0.174 and 0.165 RFU (Fig. 2(a, e)). The simulated RFU of all labelling time points and peptides agrees well with the experimental data with no significant discrepancies in the gradient of the fit between these data (Fig. 2(b, f)). While the native state HDX-MS simulations of both proteins compare equally well with their respective experimental outputs, there are significant differences in their overall diagnostic ability. For a set of 1000 protein decoys, there are many native (low RMSD) alpha lactalbumin structures that also yield HDX-MS simulations that align closely with the experimental outputs (low RMSE). This contrasts with the barnase decoy set where the clustering around native structures that also generates accurate HDX-MS simulations is qualitatively less apparent (Fig. 2(c, g)). Differences in the ability of HDX-MS to discriminate between native and non-native protein folds of these proteins were confirmed from the associated ROC plots. The alpha lactalbumin and barnase data have respective AUC values of 0.96 and 0.85 indicating that the HDX-MS simulations of alpha lactalbumin are > 3-fold more likely to correctly identify native and non-native structures than those of barnase (Fig. 2(d, h)). Differences in the diagnostic ability of the HDX-MS of these proteins could reflect variations in the number of peptides that comprise each dataset. While both proteins have similar chain lengths, the barnase HDX-MS profile is comprised of around 50% fewer peptides. Despite a significant region of missing peptides around two of the disulphide bonds of alpha lactalbumin, the peptide redundancy is significantly higher for this protein. High redundancy may enhance the ability of the alpha lactalbumin HDX-MS data to discriminate between different folds resulting in the exceptionally high AUC (Fig. 3).

The accuracy of the HDX-MS simulations of these proteins is remarkable given that the underlying PF estimates correlate poorly with previously determined experimental values (Fig. S1). The HDX-MS data were also simulated directly from crystal structures of the proteins which neglect the ensemble property of HDX and the understanding that exchange is driven by protein motion. The coefficients β_C_, β_H_ (Eq. 1) were previously found by fitting experimental PFs from a limited number of proteins to structural ensembles generated by molecular dynamics (MD) simulations [5]. Surprisingly, however, we found that PFs simulated from the ensemble average of alpha lactalbumin corresponded less well with the experimental PFs of this protein. HDX-MS data simulated from the ensemble average also compared less well with experimental outputs (Fig. S2). Overall, PFs simulated from an MD ensemble of alpha lactalbumin reduced the accuracy of the HDX-MS simulations. While these results are somewhat unexpected, they agree with recent observations showing that data simulated from single structures can improve the correlation with experimental HDX data [25].

**Figure 4:**
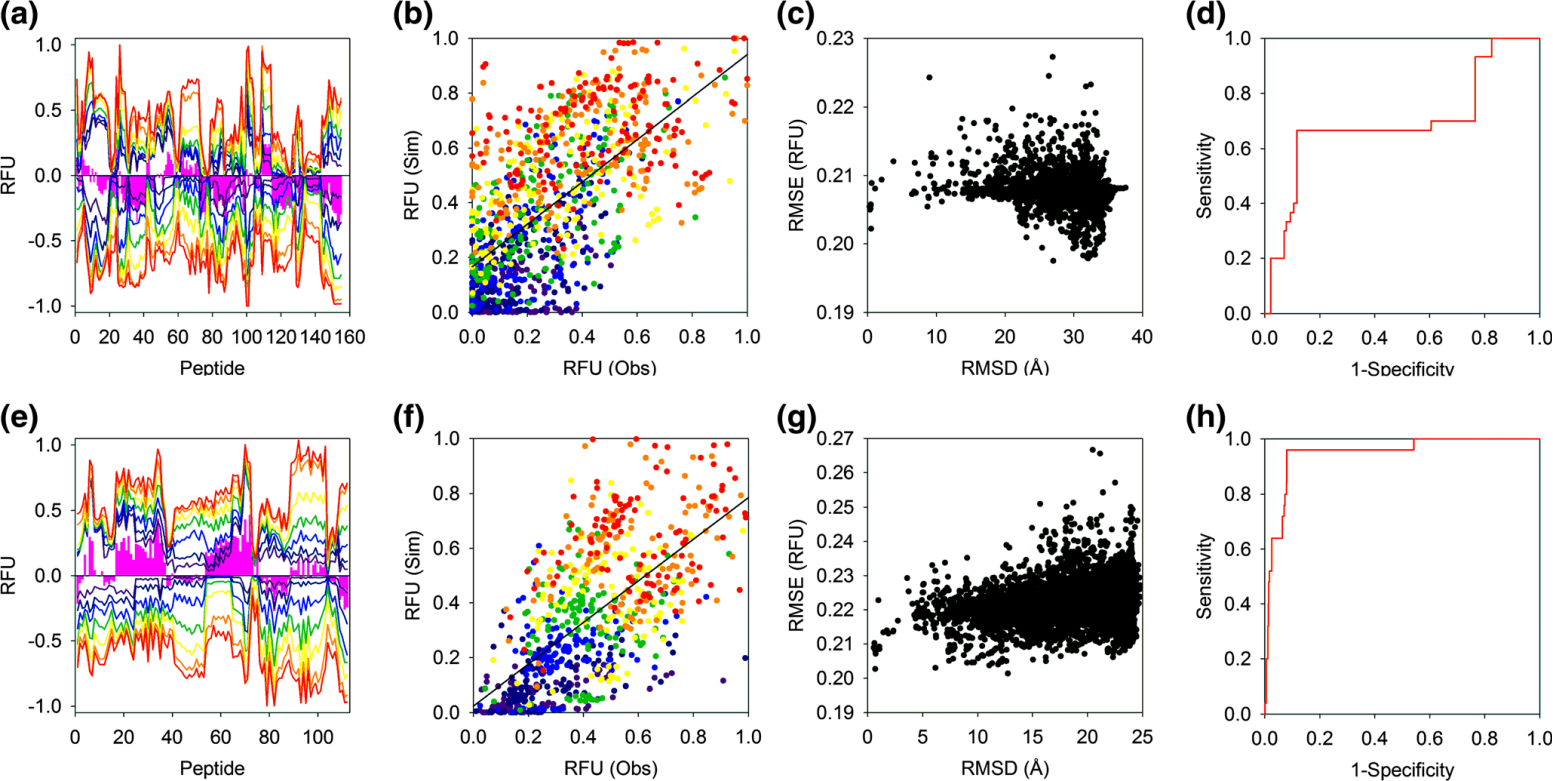
Native structures of enolase and SAP investigated by HDX-MS: (**a**, **e**) Mirror plots comparing experimental (positive) and simulated (negative) HDX-MS outputs. Experimental data were acquired at 0.25, 1, 5, 20, 60, 240, and 480 min at 293.15 K (coloured dark blue through red respectively). The pink bars denote the time-averaged difference in RFU between the experimental and simulated data and are shown to highlight areas of significant change. (**b**, **f**) Scatterplot comparing observed and simulated HDX-MS data of all RFU time points with different labelling times coloured as in (**a**). (**c**, **g**) The relationship between the RMSE and RMSD for a range of decoys. The RMSE was calculated by pairwise comparison of the simulated and experimental HDX-MS data and the RMSD determined by alignment with the crystal structure. (**d**–**h**) ROC plots demonstrating the ability of the HDX-MS simulations to classify protein structures. Decoys with an RMSD ≤ 2.5 Å with the crystal structure were classified as native. Enolase and SAP data are shown in the upper and lower four figures, respectively

We then applied the same approach to characterise the structures of the homo-protein assemblies enolase and SAP. Here, we assume the native fold of the proteins and investigate the ability of the HDX-MS simulations to identify the native chain organisation. In contrast to the HDX-MS simulations of the protein monomers, those obtained for the native protein complexes are characterised by an overall lack of correspondence with their respective experimental outputs (Fig. 4(a, e)). The HDX-MS simulations fail to broadly capture the experimental data with RMSE for the respective HDX-MS simulations of enolase and SAP of 0.219 and 0.212 RFU. The correspondence between all peptides and time points is also asymmetrical with the RFU of the simulations either under or overestimating the experimental values (Fig. 4(b, f)). Despite the poor accuracy of the HDX-MS simulations of both protein complexes, there are significant differences in their ability to discriminate between native and non-native structures. The ability of the enolase simulations to identify native structures is poor with the associated ROC plot indicating failure with an AUC of 0.69 (Fig. 4(c, d)). In contrast, however, the ability of the SAP HDX-MS simulations to correctly classify structures is extremely high with the AUC if the associated ROC plot indicating a success rate of 95% (Fig. 4(g, h)).

**Figure 5:**
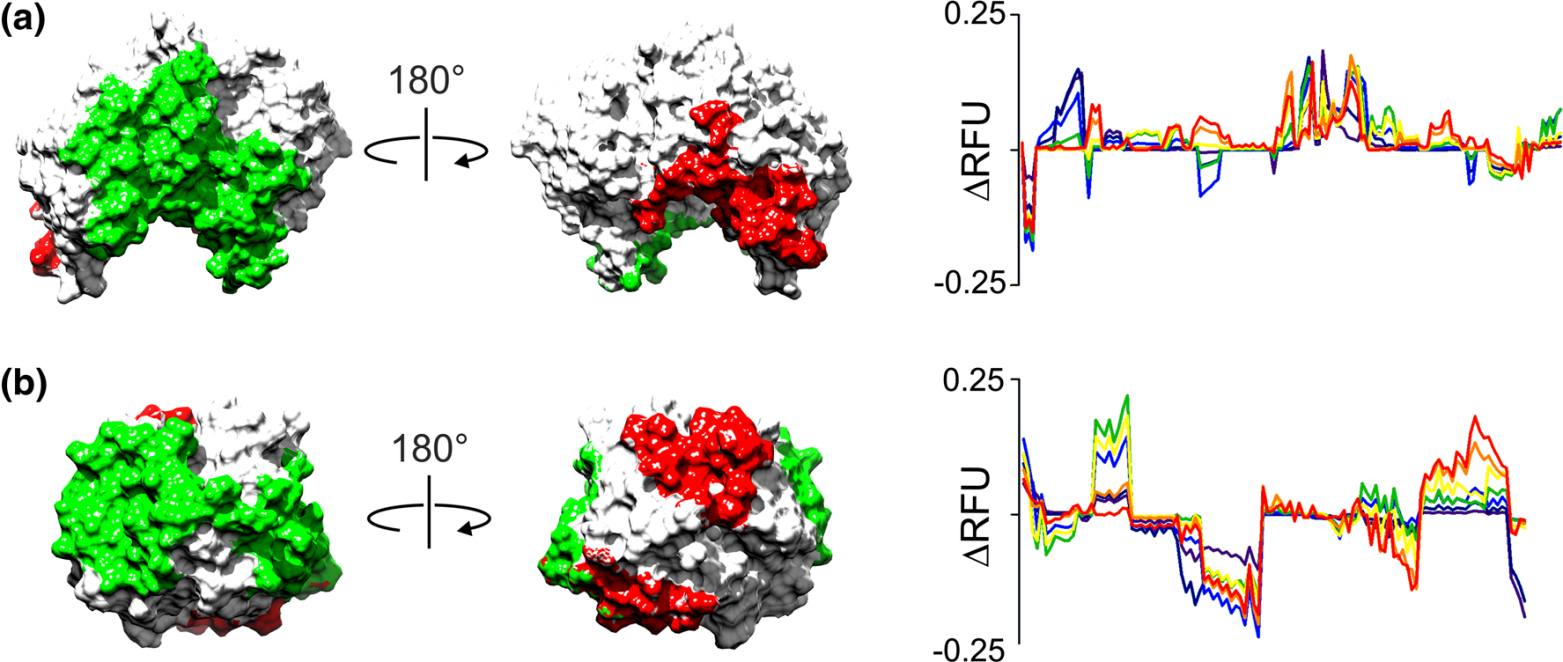
**∆**RFU for different chain orientations of enolase and SAP: (**a**) native (green) and non-native (red) protein-protein interfaces shown on a single enolase protein chain. Interfacial regions were defined using a 6.5-Å distance cutoff as used in Eq. 1. The plot shows the ∆RFU between the native and non-native assembly for all peptides. (**b**) as per (**a**) but shown for SAP the ∆RFU between the native and non-native SAP assemblies for all peptides is also shown. Data in the ∆RFU plots reflect the seven different labelling times from 15 s to 8 h, coloured dark blue to red respectively. Non-native interfaces for both proteins represent assemblies with the highest RMSD after alignment with the native complex

Given the inaccuracy of the HDX-MS simulations of both enolase and SAP, the high diagnostic ability of the SAP simulations is unexpected. This is likely attributed to differences in the number of interchain contacts in these proteins. Whereas a significant proportion of each SAP monomer is buried in subunit interfaces of the pentameric complex, the buried regions of each enolase chain are limited to a single dimeric interface. Accordingly, the likelihood of peptides probing protein-protein interfaces is much higher in SAP such that the HDX-MS outputs of this complex can more effectively differentiate between different chain orientations. To highlight this, HDX-MS data were simulated for both enolase and SAP showing the change in RFU (∆RFU) between the native and a non-native protein complex. As expected, the proportion of each protein chain buried in protein-protein interfaces is significantly higher in SAP with the consequence that many more SAP peptides exhibit large changes in their RFU for the different subunit poses and the ∆RFU of the SAP peptides is more widespread and pronounced (Fig. 5). We suggest that the increased number of interchain contacts in SAP enhances the ability of the HDX-MS simulations of this protein to discriminate between different assembly structures. High numbers of interchain contacts must therefore be particularly important for the modelling of homo-protein complexes by HDX-MS and may in some cases overcome limitations in the accuracy of the simulated data.

## Conclusion

The aim of this work was to quantify the ability of HDX-MS to discriminate between native and non-native protein conformations based on a popular approach to estimate PFs from protein structures. The efficacy of the method was evaluated on the peptide level using the PF estimates to calculate HDX-MS outputs of proteins and their assemblies and then comparing these simulations to experimental data obtained in-house. The ability of HDX-MS to identify native structures was quantified based on their performance in binary structural classification to provide insight into the use of HDX-MS for protein modelling.

We show that HDX-MS data simulated directly from protein atomic structures can be highly diagnostic for native protein folds, even when the underlying PFs of these data are poorly defined. For alpha lactalbumin, PF calculations (lnP) with an RMSE of only 2.86 over 44 residues were sufficient to generate HDX-MS outputs capable of discriminating between native and non-native states with a success rate of > 95% (Fig. S1). Our data suggest that high-peptide redundancy may be more important than overall coverage in the ability of HDX-MS to differentiate between native and non-native structures. The alpha lactalbumin HDX-MS data significantly outperformed that of barnase in binary structural classification despite having a peptide coverage of only 82% compared with 99% for barnase. Although the native state HDX-MS simulations of both these proteins agreed equally well with their respective experimental profiles, the peptide redundancy of the alpha lactalbumin data is significantly higher. We propose that the high-peptide redundancy of the alpha lactalbumin HDX-MS outputs enhances the capacity of these data to differentiate between different folds resulting in the exceptionally high AUC. Remarkably, protein ensembles were not required for these calculations and even reduced the accuracy of the simulated protection factors. While this observation contradicts accepted relationships between protein motions and exchange behaviour, the capacity to generate accurate HDX-MS data from unique states is appealing because of the associated benefits with regard to throughput.

HDX-MS data simulated for homo-protein assemblies compared significantly less well with experimental outputs. This could be due to significant differences in the HDX behaviour of protein complexes and the fact that Eq. 1 was never optimised for use with large multi-chain proteins. To better understand the scope of Eq. 1, HDX-MS data were simulated over a range of different *β*_C_, *β*_H_ weighting values and the outputs compared the experimental data. While the expression could be marginally optimised to improve the correspondence between the simulated and experimental profiles, this did not improve the ability of the simulations to correctly classify the quaternary conformations of protein assemblies (Fig. S3). The inability of Eq. 1 to describe the HDX behaviour of protein assemblies may originate from more pronounced EX1 exchange in these assemblies which is not defined by the current approach. However, no significant EX1 signatures were visible in the experimental isotope patterns of these proteins suggesting that equilibrium exchange (EX2) dominates the isotope uptake of these proteins (data not shown). Interestingly, the HDX-MS simulations of the pentameric protein assembly SAP were shown to be highly diagnostic of the native complex in spite of their poor correspondence with experimental data. We suggest that this stems from a greater number of protein-protein interfaces in this complex with an associated increase in the number of peptides available to sample native and non-native chain orientations. However, this observation also points to a limitation in the characterisation of homo-protein complexes in that knowledge of peptide redundancy and coverage in the native interface can only be had with the aid of a high-resolution structure. This is not a challenge for hetero-proteins however, as the degree of peptide sampling in the native interface can be inferred directly from associated HDX-MS difference data without the need for any structural reference. Indeed, the ability of HDX-MS to provide detailed footprinting information on the protein-protein interfaces of hetero-protein complexes in the absence of any structural information is one of the major strengths of the technique.

We have demonstrated that a simple expression used to calculate protein exchange behaviour is sufficient to simulate HDX-MS data that can effectively differentiate between native and non-native protein folds. While these data are limited to a few selected protein structures and further work is required to understand the scope of these expressions, they do provide an important window in the use of HDX-MS for protein modelling. Peptide redundancy appears to be more important than overall coverage for these approaches and a high degree of interchain contacts is essential for HDX-MS guided modelling of protein complexes. Future work to characterise and develop improved expressions for calculating the PFs of proteins from their atomic structures may unlock previously untapped potential of HDX-MS in areas such as ab initio protein folding and high-throughput structure determination. This will require a greater understanding of the relationship between protein structure and HDX for which the present work represents a useful platform.

## Electronic supplementary material

ESM 1 (DOCX 2596 kb)
