# Supplementary material for: Quantitative Evaluation of Native Protein Folds and Assemblies by Hydrogen Deuterium Exchange Mass Spectrometry (HDX-MS)
Source: J Am Soc Mass Spectrom. 2018 Oct 2;30(1):58–66. doi: 10.1007/s13361-018-2070-3 (PMC6318237; doi:10.1007/s13361-018-2070-3)
Supplement: Supplementary file 1 — (DOCX 2596 kb) [file 13361_2018_2070_MOESM1_ESM.docx]

**Quantitative evaluation of native protein folds and assemblies by hydrogen deuterium exchange mass spectrometry (HDX-MS)**

Matthew J Harris, Deepika Raghavan and Antoni J Borysik*

Department of Chemistry, King’s College London, Britannia House, London SE1 1DB, United Kingdom

**Supporting information**

**Optimisation of simulated protection factors:** Equation 1 was used to estimate the HDX protection factors (PFs) of proteins from their atomic coordinates, where the protection of residue *i* is expressed as the number of heavy atoms ($N_{i}^{C}$) and hydrogen bond acceptors ($N_{i}^{C}$) within defined distance cut-offs of the backbone amide each scaled by an empirically defined weighting term (*β*). Protection factors simulated from the crystal structure of human alpha lactalbumin were found to be significantly higher than previously determined experimental values. [1] To bring these data in line a separate exclusion parameter was introduced which reduced the number of atoms in the contacts calculations and consequently the overall PFs (Figure S1). In Equation 1 protection factors are estimated from a discrete count of contacts within the cutoff distance. To further reduce the value of the estimated protection factors a smooth function was also introduced to count these atoms (Equation 2). Overall an exclusion parameter of 3 with smoothed counting of contacting residues resulted in the highest correlation between the experimental and simulated PFs of alpha lactalbumin with an R^2^ coefficient of 0.173 lnP (Figure S1).


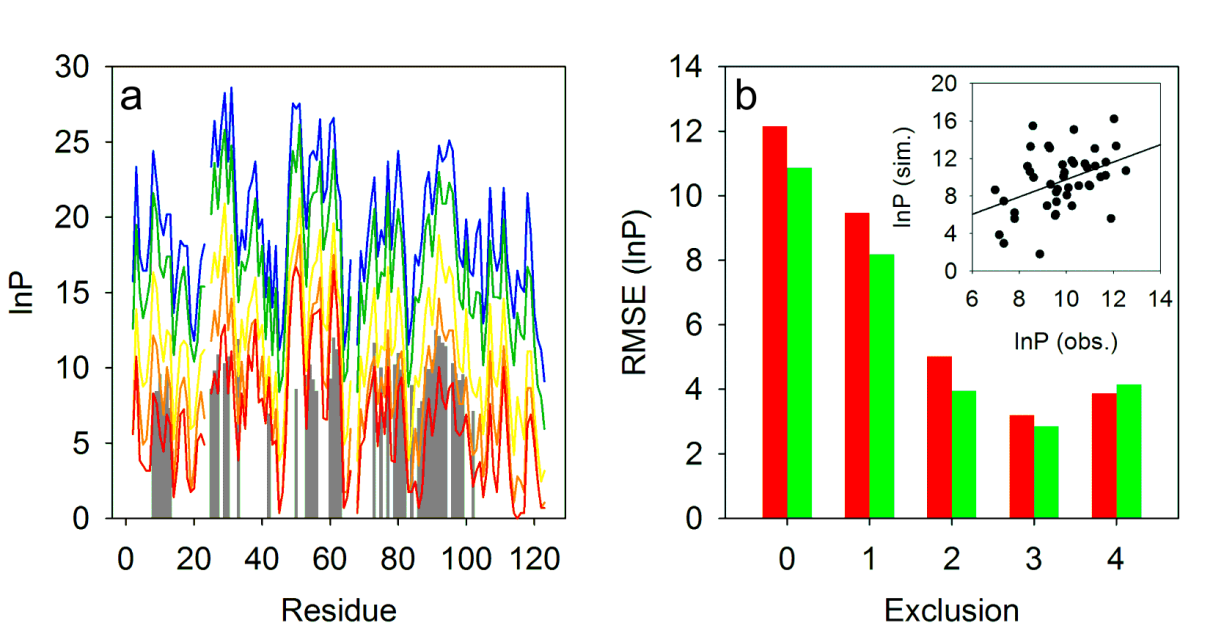


**Figure S1 Optimisation of simulated protection factors:** **(a)** Simulated (coloured lines) and experimental (grey bars) protection factors of human alpha lactalbumin. The simulated values were obtained using previously defined values of β_C_, β_H_ and calculated at exclusion parameter values of 0, 1, 2, 3, and 4 for the respective blue to red traces. **(b)** Dependence of the RMSE between the simulated and experimental protection factors of alpha lactalbumin as a function of the exclusion parameter. Protection factors were calculated using discrete (red bars) or smooth (green bars) counting of the contacting atoms. The correlation between the experimental protection factors and the simulated values from the optimised equation are shown and has an associated R^2^ coefficient of 0.173 (insert). Protection factors are given as natural log values (lnP).

**HDX-MS simulations of alpha lactalbumin from MD ensembles:** Protection factors were also taken from the ensemble average of a 100 ns MD simulation of alpha lactalbumin in explicit water at 300K using the expression optimised for the crystal structure (see above). The correlation between the experimental and simulated PFs for the MD ensemble was 0.162 compared with 0.173 for PFs simulated for the crystal structure. HDX-MS outputs simulated using the MD ensemble PF averages correlated less well the experimental data with an RMSE of 0.21 due mainly due to a large overestimation of exchange rates between peptides 20 – 30. The linear trend between the RFU of all peptides and time points was also less ideal than for the crystal structure with a corresponding R^2^ coefficient of 0.61 (Figure S2). Overall lnP simulated from the ensemble average of alpha lactalbumin had a negative effect on the correlation between experimental and simulated HDX-MS data.

**
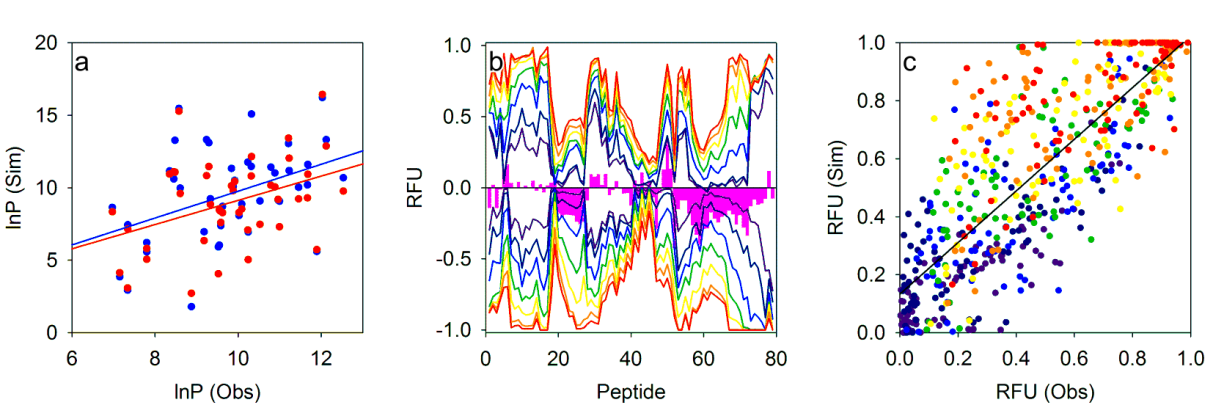
**

**Figure S2 HDX-MS simulations of alpha lactalbumin from MD ensembles: (a)** Correlation between the experimental PFs and values simulated from the crystal structure (blue) and MD ensemble (red) of alpha lactalbumin. **(b)** Mirror plot comparing experimental (positive) and simulated (negative) HDX-MS patterns of alpha lactalbumin. Experimental data were acquired using a continuous labelling workflow at 0.25, 1, 5, 20, 60, 240 and 480 minutes at 293.15 K (coloured dark blue through red respectively). The simulated RFU were taken from the ensemble average PFs of alpha lactalbumin. The pink bars denote the time averaged difference in RFU between the experimental and simulated data and are show to highlight areas of significant change. **(c)** Scatterplot comparing observed and simulated HDX-MS data over all RFU time points with different labelling times coloured as in **(a)** the R^2^ coefficient between the data is 0.61.

**Optimisation of simulated HDX-MS patterns:** To understand the scope of Equation 1 and ascertain to what degree the simulated HDX-MS outputs could be optimised for each protein, data were simulated over a range of β_H_, β_C_ weighting terms and then compared to experimental outputs. While varying the weighting terms improved the RMSE between the experimental and simulated HDX-MS data for all proteins the effect was marginal with the greatest improvement being for enolase with a reduction in the RMSE of 0.05 RFU (Figure S3a – d). Optimisation of Equation 1 did bring about qualitative improvements in the native state HDX-MS simulations particularly for the protein assemblies (Figure S3e – h). The gradients of the fits between the experimental and simulated data for all peptides and time points were also marginally improved particularly for the enolase (Figure S3i – l). The ability of the optimised expressions to classify protein structures was then investigated. A new library of HDX-MS simulations was prepared for each decoy set using the optimised expressions and compared to the respective experimental outputs (Figure S3m – p). Optimisation of the expressions did not result in any significant improvement in the diagnostic ability of the simulations with the greatest change in AUC being for alpha lactalbumin HDX-MS from 0.96 to 0.97. Surprisingly, for the protein complexes optimisation of Equation 1 was actually detrimental on the capacity of the HDX-MS simulations to classify structures. The loss of diagnostic ability was most pronounced for enolase with a reduction in the AUC from 0.69 to 0.60 (Figure S3q – t). To understand this further the percent change in the RMSE for each decoy upon optimisation of Equation 1 was calculated and plotted against the respective RMSD of each structure. For the protein assemblies this revealed that optimisation of the β_H_, β_C_ weighting terms for the native structure preferentially optimised the HDX-MS simulations of non-native states. The RMSE of the HDX-MS simulations of non-native structures was improved more significantly than those generated for more native decoys (Figure S3u – x). This reveals that optimisation of Equation 1 for native structures does not necessarily result in improvements in their classification ability and can in some cases have a detrimental effect on the capacity of the simulations to differentiate between native and non-native states (Figure S3u – x). Overall these data suggests that it may not be possible to sufficiently optimise Equation 1 for protein complexes and further work will be required to develop expressions that can better capture the HDX behaviour of these systems.


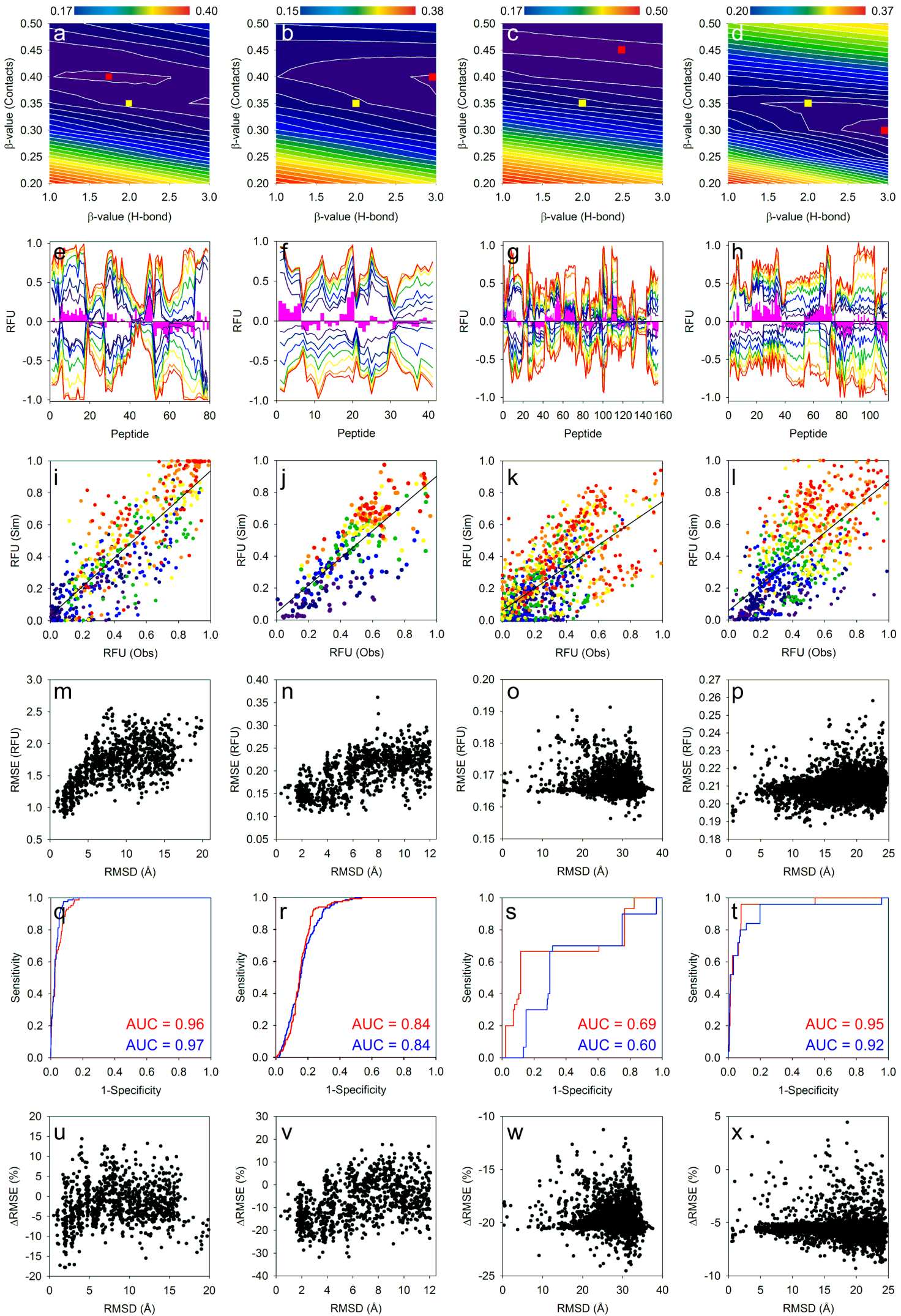


**Figure 4 optimisation of simulated HDX-MS data: (a – d)** Contour plots showing the RMSE (RFU) between experimental and simulated HDX-MS data as a function of the β_H_, β_C_ weighting terms. The original and optimised positions are indicated by the respective yellow and red squares with the RMSE legend given above each plot. The contour plots are the average RMSE taken across all labelling times. **(e – h)** Mirror plots showing experimental (positive) and simulated (negative) HDX-MS outputs. The simulated data was generated using the optimised expressions. Labelling times are at 0.25, 1, 5, 20, 60, 240 and 480 minutes at 293.15 K (coloured dark blue through red respectively). The pink bars denote the time averaged difference in RFU between the experimental and simulated data and are show to highlight areas of significant change. **(i – l)** Scatterplot comparing observed and simulated HDX-MS data of all RFU time points with different labelling times coloured as in **(e – h)**. **(m – p)** Relationship between the RMSE and RMSD of protein decoys. The RMSE was calculated by pairwise comparison of the simulated and experimental HDX-MS data using the optimised expressions and the RMSD determined by alignment with the crystal structure. **(q – t)** ROC plots demonstrating the ability of the HDX-MS simulations to classify protein structures. Decoys with an RMSD ≤ 2.5 Å with the crystal structure were classified as native. Plots are shown at the original (red) and optimised (blue) β-values and the AUC for each plot is shown. **(u – x)** The percent change in RMSE of each decoy upon optimisation of Equation 1 plotted as a function of their RMSD obtained by alignment with the native state. Data are shown for proteins in each column reading from right to left for alpha lactalbumin, barnase, enolase and SAP.

1. Schulman, B.A., Redfield, C., Peng, Z.Y., Dobson, C.M., Kim, P.S.: Different subdomains are most protected from hydrogen exchange in the molten globule and native states of human alpha-lactalbumin. Journal of molecular biology. **253**, 651-657 (1995)
